# A Pilot Epigenome-Wide Study of Posttraumatic Growth: Identifying Novel Candidates for Future Research

**DOI:** 10.3390/epigenomes9040039

**Published:** 2025-10-06

**Authors:** Mackenzie Rubens, Paul Ruiz Pinto, Anita Sathyanarayanan, Olivia Miller, Amy B. Mullens, Dagmar Bruenig, Patricia Obst, Jane Shakespeare-Finch, Divya Mehta

**Affiliations:** 1 Centre for Genomics and Personalised Health, Queensland University of Technology (QUT), Kelvin Grove, QLD 4059, Australia; e.rubens@qut.edu.au (M.R.); paul.ruizpinto@qut.edu.au (P.R.P.); anita.sathyanarayanan@connect.qut.edu.au (A.S.); d.bruenig@qut.edu.au (D.B.); 2 School of Biomedical Sciences, Queensland University of Technology (QUT), Brisbane, QLD 4000, Australia; 3 Centre for Data Science, Queensland University of Technology (QUT), Brisbane, QLD 4000, Australia; 4 School of Psychology and Counselling, Queensland University of Technology (QUT), Kelvin Grove, QLD 4059, Australia; o3.miller@qut.edu.au (O.M.); p.obst@qut.edu.au (P.O.); j.shakespeare-finch@qut.edu.au (J.S.-F.); 5 School of Psychology and Wellbeing, University of Southern Queensland (USQ), Toowoomba, QLD 4350, Australia; amy.mullens@usq.edu.au

**Keywords:** posttraumatic growth, posttraumatic stress disorder, stress, EWAS, DNA methylation

## Abstract

Background: Posttraumatic growth (PTG) refers to positive psychological change following trauma. While its psychological aspects are well-documented, the biological mechanisms remain unclear. Epigenetic changes, such as DNA methylation (DNAm), may offer insight into PTG’s neurobiological basis. Aims: This study aimed to identify epigenetic markers associated with PTG using an epigenome-wide association study (EWAS), the first of its kind in a trauma-exposed population. Methods: A longitudinal EWAS design was used to assess DNAm before and after trauma exposure in first-year paramedicine students (n = 39). Genome-wide methylation data were analyzed for associations with PTG, applying epigenome-wide and gene-wise statistical thresholds. Pathway enrichment analysis was also conducted. Results: The study identified two CpGs (cg09559117 and cg05351447) within the PCDHA1/PCDHA2 and PDZD genes significantly associated with PTG at the epigenome-wide threshold (*p* < 9.42 × 10^–8^); these were replicated in an independent sample. DNAm in 5 CpGs across known PTSD candidate genes ANK3, DICER1, SKA2, IL12B and TPH1 were significantly associated with PTG after gene-wise Bonferroni correction. Pathway analysis revealed that PTG-associated genes were overrepresented in the Adenosine triphosphate Binding Cassette (ABC) transporters pathway (*p* = 2.72 × 10^−4^). Conclusions: These results identify genes for PTG, improving our understanding of the neurobiological underpinnings of PTG.

## 1. Introduction

Posttraumatic growth (PTG) describes both the process of positive psychological change resulting from exposure to extreme challenges, as well as the resulting improvements across varied domains of psychological functioning [1] These domains include interpersonal relationships, perceptions of personal strength, appreciation for life, and spiritual and existential beliefs [2]. PTG is common following trauma exposure and represents important psychological processes of the interaction of ongoing stress resulting from the exposure to a traumatic event and positive trajectories afterwards [3,4]. For example, Vietnam War veterans who had been prisoners of war reported positive outcomes resulting from their experience, including increased optimism, social support, and adaptive coping [3]. The capacity to grow and adapt can enable individuals to develop new skills and thrive following traumatic experiences.

The model of PTG conceptualises trauma as a challenge to an individual’s core beliefs. The process of PTG is then the cognitive processing and resolution of this challenge and the eventual integration of this resolution into new beliefs [5]. In contrast, posttraumatic stress disorder (PTSD) is the ongoing conflict experienced by an individual who has not resolved the challenge to their beliefs that was presented by the traumatic experience. The relationship between exposure to trauma and negative sequelae is well established, with PTSD having a lifetime prevalence of between 0.5–9% in adult Western populations [6,7]. Epidemiological studies estimate that 8–12% of adults who experience a traumatic event develop PTSD [8]. Despite PTG being more common as a posttraumatic outcome [3,4], PTSD has been the predominant focus of genomics research [9].

The processes of PTG and PTSD are not mutually exclusive and have been shown to co-occur following trauma exposure [10,11]. The nature of the relationship between the two outcomes has remained ambiguous, with studies suggesting a significant positive relationship between symptoms of PTSD and PTG [12], a significant negative relationship [13], or no relationship at all [14]. A meta-analysis of 42 papers within populations of varied backgrounds, ages, and trauma types found that a curvilinear model was a significantly stronger predictor of the relationship between PTG and PTSD symptoms [15]. The relationship was affected by age at the time of exposure, with children fitting the curvilinear model more strongly than adults, and type of trauma. This meta-analysis represents one of the largest attempts at quantifying the relationship between PTSD and PTG. An approach that has only begun to emerge in recent years has involved the measurement of biological factors and underlying genetics as possible drivers of differences in posttraumatic outcomes, especially PTSD versus PTG.

Genetic factors have been well-established as contributing to the development of PTSD following trauma exposure [16,17]. The effect of genetics on PTG, however, is comparatively under-researched [18]. The gene–environment interaction (GxE) in a population of non-Hispanic African American parents exposed to a natural disaster was the first to include an assessment of PTG [19]. The study explored whether common variants of seven genes (*BDNF*, *CACNA1C*, *CRHR1*, *FKBP5*, *OXTR*, *RGS2*, and *SLC6A4*) modified the association between Hurricane Katrina exposure, PTSD, and PTG. A nominally significant association was found between a variation in *FKBP5* and PTG that did not survive correction for multiple testing (rs1306780, *p* = 0.0113). Additionally, a significant association was found between a variant of the *RGS2* gene and PTG that did survive correction for multiple testing (rs4606; *p* = 0.0044). This variant interacted with the severity of trauma exposure such that individuals with low levels of exposure showed PTG scores, and individuals with moderate or high levels of exposure showed increased levels of PTG. This *RGS2* variant had been shown to moderate the association between trauma severity and PTSD in a previous study, with decreased levels of PTSD symptom severity [20]. The *RGS2* gene codes for a protein that regulates G-protein signalling and modulates neurotransmitter response, with different variants of this gene accelerating the deactivation of G-proteins at different rates [21].

While the DNA code remains stable over the lifespan, epigenetic processes, such as DNA methylation (DNAm), are dynamic and can be affected by different cellular environments and lived experiences. DNAm involves the addition of a methyl-chemical group to specific locations within the genome, which usually blocks access of transcription factors to the DNA, resulting in reduced expression of that gene downstream [22]. Trauma exposure has been associated with alterations in DNAm in epigenome-wide association studies (EWAS) [23] as well as studies of specific candidate genes [24]. An EWAS in Australian veterans identified DNAm at *DOCK2*, a gene involved in the formation of amyloid plaques in Alzheimer’s disease [25], to be associated with PTSD [26], highlighting the importance of memory processes in post-trauma trajectories. A separate study examined DNAm before and after combat exposure in a cohort of male US military service members and found associations between PTSD and altered DNAm at *HEXDC* and *MADL1* genes, suggesting the involvement of immune pathways [27].

Only one study has explored the association between PTG and DNAm [28]. In a sample of 48 first-year paramedicine students, PTSD symptom severity, resilience, and PTG were associated with DNAm levels in candidate genes *FKBP5* and *NR3C1* [28]. Specifically, hypomethylation (reduced methylation) at the CpG site cg07485685 within *FKBP5* was associated with increased PTSD symptom severity, while hypermethylation (increased methylation) was associated with resilience. Differential DNAm in multiple sites across *FKBP5* and *NR3C1* were associated with PTG, though these associations did not survive Bonferroni corrections for multiple testing.

In summary, the research on PTG thus far has only been cross-sectional in nature and has focussed on specific candidate genes. This study employs a longitudinal design to assess genome-wide changes in DNAm and their association with changes in PTG scores following exposure to a traumatic event. The aim of the study was to identify which genes and pathways are associated with PTG and compare the genes to those associated with PTSD, to uncover the genetic etiology of PTG.

## 2. Results

A total of 39 first-year paramedicine students at two Australian universities were included in the study. Psychological data via online surveys and DNAm via saliva samples was measured at two time-points—before (T_1_) and after (T_2_) exposure to potentially traumatic events. Study demographics are provided in Table 1. The participants were predominantly females (61.5%), Caucasian (89.7%), and with a mean age [SD] of 23.44 [1.08] years. In the current study, PTG and PTSD symptom severity were not significantly correlated at T_1_ (Spearman correlation *r* = 0.252, *p* = 0.122) or T_2_ (Spearman correlation *r* = 0.140, *p* = 0.402). There was a significant decrease in PTG scores from T_1_ and T_2_ (*p* = 0.032). There was a significant decrease in the overall PTSD PCL-5 score from T_1_ to T_2_ (*p* = 0.029) which was mainly driven by change in the sub-scale assessing cluster D symptoms of negative alterations in cognition and mood (*p* = 0.004). All other sub-scales showed non-significant differences between T_1_ and T_2_ (*p* > 0.05).

Although PTG is often conceptualised as a positive trajectory following trauma, early elevations may reflect short-term adaptive coping or cognitive reframing that naturally recalibrates as individuals gain psychological clarity over time [5]. The observed decrease in PTG scores may therefore represent a shift from initial perceived growth to a more integrated and realistic appraisal of the trauma experience. Simultaneously, reductions in PTSD symptoms particularly in cognitive and mood-related domains may reflect the influence of protective psychosocial factors such as social support and belongingness, which have been shown to buffer distress and promote recovery [3,29]. These findings align with broader evidence suggesting that biological and environmental interactions, including epigenetic regulation, may contribute to individual variability in post-trauma adaptation [19,24].

### 2.1. Candidate Gene Analysis

This is the first epigenome-wide analyses of PTG; therefore, as a proof of principle, genes previously associated with PTSD were first tested to ascertain if these were also associated with PTG. Specifically, changes in PTG from T_1_ to T_2_ were tested for their association with DNAm changes in 55 candidate genes previously associated with PTSD [26]. Of the 3811 CpGs across 53 of the PTSD genes present in this dataset, 236 CpGs across 47 genes were nominally associated with changes in PTG scores (*p* < 0.05). Of these, 5 CpGs across five genes remained significant after a gene-wise Bonferroni correction, this is significantly greater than expected by chance alone (enrichment *p*-value = 0.003, Table 2). The genes included ankyrin3 (*ANK3)*, dicer 1, ribonuclease III (*DICER1)*, spindle and kinetochore associated complex subunit 2 *(SKA2)*, interleukin 12B (*IL12B)* and tryptophan hydroxylase 1 (*TPH1)*. Given the small sample size, the candidate gene enrichment analysis is exploratory and should be interpreted with caution.

### 2.2. EWAS of PTG

**Figure 1:**
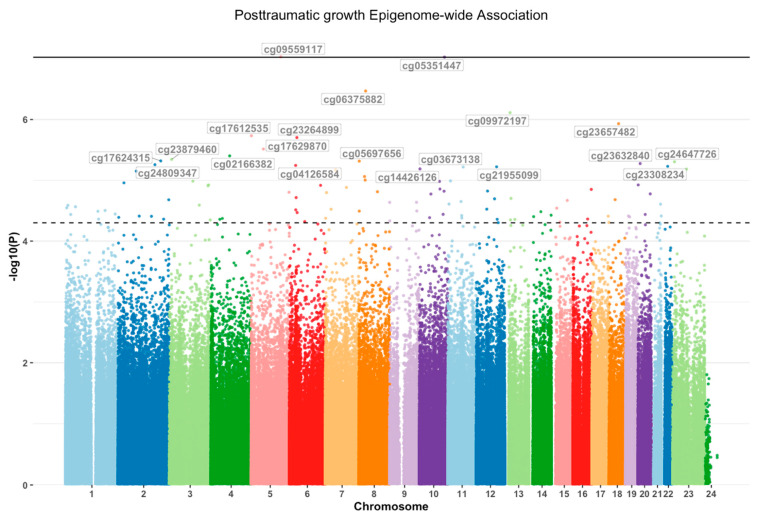
PTG associations: Manhattan plot showing epigenome wide DNAm associations for changes in PTG after trauma exposure. The epigenome-wide threshold (*p* < 9.42 × 10^−8^) is indicated by the bold line, and the suggestive threshold of significance (*p* < 5 × 10^−5^) is indicated by the dotted line.

A hypothesis-free epigenome-wide analysis was performed to identify changes in DNAm associated with changes in PTG across the two time-points (before/T_1_ and after exposure to a traumatic event/T_2_). Across the 845k CpGs assessed, two CpGs were significantly associated with changes in PTG between T_1_ and T_2_ even after correction at the epigenome-wide level [30]. The significant sites included cg09559117 in *PCDHA1/PCDHA2* (*p* = 9.28 × 10^−8^) and cg05351447 in *PDZD8* (*p* = 9.39 × 10^−8^, Figure 1, Supplementary Table S1). To replicate these findings, we investigated a demographically matched sample of 51 first-year university students before and after exposure to a highly stressful event. These samples were run on the latest EPICv2 arrays; hence, we investigated the probes closest to the top EWAS hits above and found that CpGs within PCDHA1 (cg05181804, *p* = 0.00032, 8.8 kb from EWAS probe), PCDHA2 (cg21619814, *p* = 0.015, 0.59 kb from EWAS probe) and PDZD8 (cg09437460, *p* = 0.047, 11.5 kb from EWAS probe) were significantly associated with changes in PTG. When using the suggestive level of significance of *p* < 5 × 10^−5^ [31], 99 CpGs across 71 genes were associated with changes in PTG scores across the two time-points (Table 3).

Next, the biological pathways of the genes associated with PTG at the suggestive level of significance (*p* < 5 × 10^−5^) and those at a less stringent significance threshold (*p <* 0.001) were assessed using the KEGG pathway database via the online WebGestalt 2024 interface [32]. The genes (n = 71) that were associated with PTG at *p* < 5 × 10^−5^ were significantly enriched in only the Adenosine triphosphate Binding Cassette (ABC) transporters pathway (*p* = 2.72 × 10^−4^*)*. The genes (n = 1150) associated with PTG at *p* < 0.001 were significantly enriched in various pathways as shown in Table 4. The top pathways included Phospholipase D signalling, Axon guidance, EGFR tyrosine kinase inhibitor resistance, morphine addiction and dopaminergic synapse pathway. While these results are of interest, given the small sample size of both the discovery and replication samples, the findings are underpowered and should be interpreted with caution until confirmed in larger studies.

### 2.3. Overlap Between PTG and PTSD

To test whether CpGs associated with PTG were also associated with PTSD, the results of the PTG epigenome-wide analysis were examined to check if these CpGs were also associated with changes in PTSD symptoms at the two time-points. At the epigenome-wide threshold, none of the CpGs associated with PTG overlapped with PTSD. Using a less stringent threshold of suggestive significance at *p* < 5 × 10^−5^, only the *PDE2A* gene was associated with both PTG and PTSD as shown in Figure 1. A total of 11 CpGs across nine genes were associated with changes in PTG at *p* < 5 × 10^−5^ and PTSD at a nominal *p* < 0.05. These included *NRG1*, *TRAK2*, *ABCA13*, *ADRA1A*, *SLC9A10*, *C10orf4*, *SNPH*, *RND1*, *FAM89B*, *RCBTB2* and *C12orf24*.

## 3. Discussion

This study represents the first longitudinal epigenome-wide study of PTG, exploring associations between changes in PTG scores and DNAm following trauma exposure in first year paramedicine students. Our findings provide further insights into the epigenetic underpinnings of PTG and establish a foundation for understanding the biological mechanisms that distinguish adaptive post-trauma responses.

The EWAS of PTG identified two CpG sites (cg09559117 and cg05351447) significantly associated with changes in PTG scores after multiple testing corrections at the epigenome-wide level (*p* < 5 × 10^−8^). Neither of the implicated genes have been previously associated with PTG, representing entirely novel findings in this field. The cg09559117 site lies within the *PCDHA1* gene body and close to the promoter of the *PCDHA2* gene. *PCDHA1* and *PCDHA2* are members of the protocadherin alpha gene cluster on chromosome five. The protocaderin proteins are calcium-dependent cell-adhesion proteins that are involved in the establishment and maintenance of specific neuronal connections in the brain [33]. Interestingly, the PCDH-alpha gene cluster lies downstream and in proximity (<6.5 Mb) to the *NR3C1* locus, a highly conserved human gene locus shown to be enriched in epigenetic changes following exposure to early life stress [34]. There are also other reports of the PCDH genes in psychiatric disorders. For example, genetic deletions in *PCDHA1* have been linked to bipolar disorder and schizophrenia [35]. Previous research has found that expression of the *PCDHA2* gene is significantly different in individuals with schizophrenia compared to healthy controls [36]. In rat models, altered expression of *PCDHA2* was identified in the brains one month after traumatic brain injury [37]. The cg05351447 site lies within the *PDZD8* gene body near the 3’UTR of the gene. *PDZD8* has been linked with PTSD in previous genomic research. For example, an allele of the *SLC18A2* gene was significantly associated with decreased expression of *PDZD8* in the dorsolateral prefrontal cortex of post-mortem brains of people with PTSD [38]. The identification of *PDZD8* in our PTG analysis suggests this gene may play a broader role in trauma-related outcomes beyond pathological responses.

Pathway analysis revealed that PTG-associated genes were significantly enriched in the Adenosine Triphosphate Binding Cassette (ABC) transporters pathway at the suggestive significance level. This pathway includes genes such as *ABCA13*, *ABCC5*, and *ABCA12*, and has been linked to mitochondrial dysfunction—a proposed therapeutic target for PTSD [39]. ABC transporters, particularly ABCB1/P-glycoproteins expressed on brain microglia, may play emerging roles in psychiatric disorders including Alzheimer’s disease [40]. At a less stringent threshold, additional pathways were identified, including phospholipase D signaling, axon guidance, and dopaminergic synapse pathways, suggesting complex neurobiological mechanisms underlying PTG.

The candidate gene analysis revealed significant associations between PTG and five genes previously linked to PTSD: *ANK3*, *DICER1*, *SKA2*, *IL12B*, and *TPH1*. This finding was significantly greater than expected by chance (enrichment *p*-value = 0.003), suggesting shared biological pathways between PTG and PTSD despite their distinct psychological manifestations. *ANK3* and *DICER1* are protein-coding genes that are associated with intellectual developmental disorder and global developmental delay, respectively [41,42]. Higher cognitive ability is associated with decreased PTSD symptom severity following trauma [29,43], and higher cognitive flexibility is linked with greater degrees of PTG [44]. As the *ANK3* and *DICER1* genes are associated with cognitive capacity and cognitive capacity influences posttraumatic outcomes, the altered DNAm at these loci associated with changes in PTG represents an interesting avenue for further research. The ankyrin 3 gene (*ANK3*) produces the ankyrin G protein that plays an integral role in regulating neuronal activity. It has generally been associated with various processes including reactivity to stress, impulse control, and memory [45] and bipolar disorder [46]. *DICER1* is an enzyme that generates mature microRNAs (miRNAs), which regulate gene expression post-transcriptionally in brain and other tissues; it is also involved in synaptic maturation and plasticity. Lower blood *DICER1* expression was reported to be significantly associated with increased amygdala activation to fearful stimuli which is a neural correlate for PTSD [47]. *TPH1* and *SKA2* genes are associated with mental illnesses, including PTSD, personality disorders, anxiety, and depression [48,49,50]. Mental illnesses are common sequelae following trauma, with symptom severity typically reducing with treatment and time. The association between differential DNAm within these genes and PTG could represent a pathway by which downstream effects develop.

When assessing the relationship between PTG and PTSD, there was little overlap in the CpGs associated with both PTG and PTSD. At the epigenome-wide level, no CpGs were associated with both PTG and PTSD after multiple testing corrections. Using a nominal *p*-value revealed only one CpG site shared between the two posttrauma outcomes. The CpG site cg03929569 is not linked to any gene but exists on an island on chromosome 13. Previous research with monozygotic twins discordant for cerebral palsy found significant differences in DNAm at cg03929569 [51].

This study has notable strengths. As the first EWAS of PTG, it provides an unbiased, genome-wide perspective that overcomes the limitations of candidate gene approaches. The longitudinal design, assessing DNAm both before and after trauma exposure, better establishes temporal relationships and accounts for the dynamic nature of epigenetic modifications. This approach provides stronger evidence for causation than cross-sectional studies.

This study has several important limitations. Foremost, the small sample size (N = 39) severely limits statistical power for epigenome-wide analyses, increasing the likelihood of both false positives and false negatives and reducing the stability of effect size estimates. Still, we were able to replicate other probes within the same genes to be associated with PTG in an independent longitudinal cohort of 51 students. As such, all molecular findings should be considered preliminary and hypothesis-generating, requiring replication in larger, independent cohorts before any biological conclusions can be drawn. In addition, pathway and enrichment analyses based on nominal associations are highly susceptible to noise in this context and should be interpreted with extreme caution. Finally, while the longitudinal design demonstrates feasibility, the results primarily serve to inform future study design rather than to provide definitive evidence of underlying mechanisms.

These findings have important implications for understanding the biological basis of resilience and adaptive responses to trauma. The identification of specific genes and pathways associated with PTG provides potential targets for interventions aimed at promoting post-traumatic growth rather than merely treating pathology. The distinct biological signatures of PTG versus PTSD suggest that promoting resilience may require different approaches than treating trauma-related disorders. Future research should focus on replicating these findings in larger, more diverse cohorts and investigating the functional roles of the identified genes in neuroplasticity and adaptive responses. Longitudinal studies tracking individuals over extended periods could provide insights into how epigenetic changes associated with PTG evolve over time and whether they predict long-term outcomes.

## 4. Methodology

### 4.1. Participants

Study details are reported in detail elsewhere [28]. Briefly, participants were 40 first-year undergraduate Australian university paramedicine students. Participants were assessed at baseline during their first semester of classes (timepoint 1) and again 12 months later after completing field placement (timepoint 2). All 40 participants reported exposure to a potentially traumatic event(s) as part of their fieldwork placement. The study was approved by the Queensland University of Technology (QUT) and the University of Southern Queensland University (USQ) Human Research Ethics Committee. All participants provided written informed consent.

### 4.2. Assessments

At both timepoints, participants reported demographic information, including age, sex, ethnicity, alcohol consumption, smoking, and drug use. At baseline (timepoint 1), participants reported whether they had ever experienced a traumatic event, a brief description, and an assessment of the severity and distress at the time. At timepoint 2, participants reported whether they had experienced a traumatic event during their placement and a description and ratings of severity and distress on a Likert scale from 0–9, with higher scores indicating high levels of perceived severity and distress. In addition, participants completed assessments of PTG and PTSD at both timepoints and provided DNA via a saliva sample collected in an Oragene kit (DNA Genotek, Ottawa, Ontario, Canada).

### 4.3. Posttraumatic Growth Inventory X

The Posttraumatic Growth Inventory X [52] (PTGI-X) consists of 25 items that assess how much positive psychological change has occurred as a result of exposure to a traumatic event. The items range from 0 (Not at all) to 5 (A very great degree), with higher scores indicating a greater level of growth. The PTGI-X has shown high reliability in US (α = 0.97), Turkish (α = 0.96) and Japanese samples (α = 0.95) [52]. The current sample also showed strong reliability (α = 0.96).

### 4.4. Posttraumatic Stress Disorder Checklist for DSM-V

The Posttraumatic Stress Disorder Checklist for Diagnostic and Statistical Manual of Mental Disorders, fifth edition (DSM-V) [53] (PCL-5) is a 20-item measure of PTSD symptom severity, with responses ranging from 0 (not at all) to 4 (extremely). Higher scores represent more severe symptoms. The measure can be interpreted by the overall summed score and interpreted via four sub-scales that correspond to criterion B, C, D, and E of PTSD in the DSM-V. The PCL-5 has displayed strong reliability and validity in US trauma-exposed student populations [54]. The current sample had strong reliability overall (α = 0.94) and within the subscales (ranging between α = 0.74 and α = 0.89).

### 4.5. Experiments

All experimental procedures have previously been described [28]. Briefly, the saliva samples were sent to the Australian Genome Research Facility and stored at −20 °C. DNA was extracted from saliva using the Qiagen kit (Hilden, Germany) and quality assessment was performed by resolution on a 0.8% agarose gel at 130 V for 60 min. Samples were bisulphite-converted using the Zymo EZ DNA Methylation kit (Irvine, CA, USA) and hybridised on the Illumina (San Diego, CA, USA) EPIC array [55] (Wockner et al., 2014). DNA for one sample did not satisfy quality standards at timepoint 2 and was removed from all further analyses, leaving 39 individuals across both time points and a total of 78 samples.

### 4.6. Statistical Analysis and Power Calculations

Data were analysed using an established analysis pipeline comprising custom statistical programs and scripts [56,57,58] written in R and Linux. Raw beta values from EPIC Illumina arrays were exported into R version 4.5.1 for statistical analysis. The raw DNAm data was background- and control-normalized using the Bioconductor MINFI package v. 1.4.0 [59]. A detection *p*-value was calculated for all arrays, where *p*-value > 0.05 indicates methylation that is not significantly different from background measurements. We used excluded probes with *p*-detection > 0.01 in 10% or more samples. Samples with probe detection call rates < 95% as well as those with an average intensity value of either <50% of the experiment-wide sample mean or <2000 arbitrary units (AU) were excluded from further analysis. This resulted in a total of 864,424 probes for all subsequent analyses. Cell counts were analysed using the Middleton method [60]. We used generalised linear mixed effects models to model the changes in DNAm at two timepoints, which we then regressed against the phenotype of interest (scores on the PCL-5 and PTGI-X). We corrected for covariates of age, sex, body mass index/BMI, cell counts, smoking, alcohol, drug use, and medication status using the lme4 package in R version 4.5.1 [61]. For the epigenome-wide analyses, the epigenome-wide threshold (*p* < 9.42 × 10^−8^) was used to identify significant sites [30], and the suggestive threshold of significance (*p* < 5 × 10^−5^) was used to denote suggestive sites of relevance [31]. For the candidate genes, multiple testing across the different outcomes was adjusted using a gene-wise Bonferroni correction for multiple results to report results of interest. The hypergeometric test was used to test for the enrichment to assess if the observation is indeed statistically significant, i.e., beyond what is expected by chance, and this was performed in R. For the pathway analysis, CpGs were first annotated to genes using the Illumina EPIC array Manifest file and then assessed via the KEGG pathway analysis through the online WEB-based GEne SeT AnaLysis Toolkit/WebGestalt interface [32] using a false discovery rate of 5% to account for multiple testing corrections.

Analysis of the psychological variables was performed in IBM SPSS Statistics tool version 28.0.1.0 (New York, NY, USA). Changes in the PTG and PTSD scores between the two timepoints was performed via paired t-tests using 1000 bootstraps. Correlations between the psychological variables were performed using the non-parametric Spearman correlations.

Within a longitudinal study design, the paired-test method employs each subject as their own control, thereby removing between-subjects variability and increasing statistical power. The within-person correlations ranged between 0.92 < *r* < 0.96, with an average Spearman correlation *r* = 0.94 (SD = 0.007). These values are significantly higher than observed in similar papers within monozygotic twins [62]. Using the EPIC array power calculator [30] (Mansell et al., 2019), over 70% of the CpG sites arrayed have more than 90% power to detect small to moderate changes in DNAm (3–6%). These estimates of power are conservative given the longitudinal study design and the high within-person correlation observed in the study. Therefore, this study is well-powered to detect the observed (3–6%) DNAm changes.

### 4.7. Replication Cohort

The replication sample comprised first year undergraduate Australian university students from the sample university as the discovery sample; this was an independent sample. Participants were assessed at baseline during their first semester of classes (timepoint 1) and again 12 months later (timepoint 2). All 51 participants reported exposure to highly stressful event(s) and described their ratings of severity and distress on a Likert scale from 0–9, with higher scores indicating high levels of perceived severity and distress. The study was approved by the Queensland University of Technology (QUT) Human Research Ethics Committee. All participants provided written informed consent. The same psychological and health surveys were administered as the discovery sample and the replication sample was matched for demographics to the discovery sample (age, sex, ethnicity, *p*-values of differences in demographics >0.05). The replication sample DNA was run on the latest DNAm EPICv2 array; therefore, the same CpG probes were not available, but replication was performed using the probe nearest to the original EWAS probe in the discovery sample. All statistical analyses were performed identically to the discovery sample. 

## 5. Conclusions

The results from this first pilot EWAS of PTG have provided further insights into the biology of PTG, implicating the *PCDHA1*, *PCDHA2* and *PDZD8* genes in the aetiology of PTG. The genes and pathways identified in this study can be used in further investigation to provide insight into the etiology of PTG and how it relates to the biology underlying PTSD. Future prospective research within larger cohorts will provide more power to identify additional genes associated with PTG. Ultimately, these findings may inform the development of targeted interventions to enhance post-traumatic growth and resilience in trauma-exposed populations.

## Figures and Tables

**Table 1:** Demographics of the 39 paramedicine students included in the study.

Demographics/Traits	Minimum	Maximum	Mean [SE]/N [%]
			Overall Sample
Age (in years)	17	43	23.44 [1.080]
Sex—Male			15 [38.5%]
- Female			24 [61.5%]
Ethnicity			
- Caucasian			35 [89.7%]
- Asian			2 [5.1%]
- African American			1 [2.6%]
- Aboriginal/Torres Strait Islander			1 [2.6%]
Body Mass index/BMI	17.1	36.2	24.88 [0.75]
Current alcohol use			28 [71.8%]
Current medication			11 [28.2%]
Current smoking			5 [12.8%]
Current drugs			1 [2.6%]
**Baseline—at start of paramedicine course**			
Posttraumatic growth Inventory Score	6	120	72.05 [4.74]
Appreciation of Life	0	5	3.48 [0.19]
Personal Strength	0	5	3.36 [0.19]
New Possibilities	0	5	2.80 [0.24]
Relating to Others.	0.43	4.86	2.82 [0.20]
Spiritual and existential change	0	4.83	2.33 [0.21]
PTSD Symptoms Score (PCL)	0	50	16.82 [2.28]
PCL cluster B score	0	18	3.56 [0.67]
PCL cluster C score	0	8	1.95 [0.36]
PCL cluster D score	0	21	6.26 [0.92]
PCL cluster E score	0	12	5.05 [0.66]
Posttraumatic growth Inventory Score	6	120	72.05 [4.74]
**Follow-up—post trauma exposure**			
Posttraumatic growth Inventory Score	10	114	64.14 [3.95]
Appreciation of Life	0.33	4.67	2.99 [0.17]
Personal Strength	0.25	4.75	3.12 [0.18]
New Possibilities	0	4.8	2.36 [0.20]
Relating to Others.	0.57	4.71	2.75 [0.17]
Spiritual and existential change	0.17	4.2	1.86 [0.19]
PTSD Symptoms Score (PCL)	0	50	12.83 [2.27]
PCL cluster B score	0	13	3 [0.59]
PCL cluster C score	0	8	1.37 [0.35]
PCL cluster D score	0	20	3.97 [0.85]
PCL cluster E score	0	15	4.49 [0.70]

**Table 2:** PTSD Candidate genes in PTG with at least 10 CpGs tested and one Bonferroni significant CpG identified.

Candidate Genes	No. of CpGs Tested	No of CpGs with *p* ≤ 0.05	Survive Bonferroni (N)
HDAC4	503	6	NO
CACNA1C	298	23	NO
RORA	237	13	NO
ANK3	160	13	YES (1)
DOCK2	106	6	NO
NOS1AP	94	12	NO
NR3C1	89	6	NO
NLGN1	86	7	NO
BDNF	84	5	NO
SLC6A3	81	8	NO
WWC1	77	5	NO
CRHR1	69	5	NO
ANKRD55	58	3	NO
NR3C2	53	7	NO
COMT	47	5	NO
DICER1	57	7	YES (1)
FKBP5	45	4	NO
HEXDC	44	1	NO
CAMKMT	44	1	NO
CRHR2	41	5	NO
DRD2	41	3	NO
ADCYAP1	40	2	NO
PDE1A	40	3	NO
MAN2C1	37	1	NO
ADCYAP1R1	36	4	NO
OXTR	36	1	NO
CNR1	35	5	NO
PRTFDC1	35	4	NO
LY9	34	4	NO
TPH2	33	2	NO
SLC6A4	31	3	NO
FOS	26	3	NO
GABRA2	26	4	NO
SLC18A2	26	1	NO
ALOX12	24	3	NO
NPY	22	1	NO
HTR1A	21	3	NO
SKA2	21	1	YES (1)
IL12B	19	2	YES (1)
RGS2	18	2	NO
DBH	18	1	NO
AIM2	16	1	NO
OPRL1	40	3	NO
ZNF626	14	2	NO
GBP1	13	1	NO
PRR11	13	2	NO
TPH1	11	2	YES (1)
Total	2999	236	5

**Table 3:** EWAS genes significantly associated with changes in PTG scores.

cpg	*p*-Value PTG	Chromosome	Basepair	Gene Symbol
cg09559117	9.28 × 10^−8^	5	140173855	PCDHA2;PCDHA1
cg05351447	9.39 × 10^−8^	10	119120604	PDZD8
cg06375882	3.39 × 10^−7^	8	32113523	NRG1
cg09972197	7.70 × 10^−7^	13	26301550	ATP8A2
cg23657482	1.17 × 10^−6^	18	45102036	
cg17612535	1.85 × 10^−6^	5	932900	
cg23264899	1.98 × 10^−6^	6	35765259	CLPS
cg17629870	3.06 × 10^−6^	5	57756980	PLK2
cg02166382	3.96 × 10^−6^	4	88496363	
cg23879460	4.52 × 10^−6^	3	10806569	LOC285370
cg17624315	4.79 × 10^−6^	2	202289200	TRAK2
cg05697656	4.83 × 10^−6^	8	1897697	ARHGEF10
cg24647726	4.95 × 10^−6^	X	11128608	HCCS
cg23632840	5.29 × 10^−6^	20	10414722	C20orf94;MKKS
cg24809347	5.52 × 10^−6^	2	174723194	
cg04126584	5.69 × 10^−6^	6	29920309	
cg23308234	5.89 × 10^−6^	22	29965207	NIPSNAP1
cg21955099	5.99 × 10^−6^	12	96005661	
cg03673138	6.04 × 10^−6^	11	72385963	PDE2A
cg14426126	6.49 × 10^−6^	10	2394012	
cg17733714	6.55 × 10^−6^	X	68114285	
cg10626169	6.73 × 10^−6^	7	48319696	ABCA13
cg18825430	7.06 × 10^−6^	2	86422958	IMMT
cg07572251	8.66 × 10^−6^	8	26688088	ADRA1A
cg00739259	9.89 × 10^−6^	8	29858411	
cg13332953	1.02 × 10^−5^	11	12003759	DKK3
cg07479253	1.03 × 10^−5^	3	111904892	SLC9A10
cg06789550	1.04 × 10^−5^	10	95462915	C10orf4
cg16745960	1.10 × 10^−5^	2	27549918	GTF3C2
cg02754380	1.19 × 10^−5^	3	186369639	FETUB
cg01858394	1.19 × 10^−5^	20	1277043	SNPH
cg14673315	1.21 × 10^−5^	6	148336294	
cg00018767	1.23 × 10^−5^	3	183693809	ABCC5
cg10714329	1.31 × 10^−5^	7	100027122	MEPCE;ZCWPW1
cg14192396	1.39 × 10^−5^	10	97416393	ALDH18A1
cg26384474	1.41 × 10^−5^	16	86702325	
cg12831349	1.50 × 10^−5^	12	52935087	
cg01399353	1.51 × 10^−5^	10	117114665	ATRNL1
cg12533940	1.54 × 10^−5^	8	88056685	CNBD1
cg13810079	1.57 × 10^−5^	5	179484006	RNF130
cg00730549	1.59 × 10^−5^	7	5430660	TNRC18
cg09887207	1.67 × 10^−5^	20	58249281	PHACTR3
cg19492498	1.68 × 10^−5^	10	54531460	MBL2
cg24478695	1.92 × 10^−5^	6	32363167	BTNL2
cg03929569	1.98 × 10^−5^	13	30689009	
cg06740227	2.01 × 10^−5^	12	86229804	RASSF9
cg14263702	2.08 × 10^−5^	18	28651637	DSC2;DSC2
cg01804434	2.09 × 10^−5^	2	240456931	
cg08343397	2.14 × 10^−5^	15	75340982	PPCDC
cg24078577	2.23 × 10^−5^	11	62160859	ASRGL1
cg09039879	2.30 × 10^−5^	9	127230734	
cg13793478	2.31 × 10^−5^	9	109039	
cg17748470	2.46 × 10^−5^	11	4969161	OR51A4
cg23107033	2.47 × 10^−5^	21	44166176	PDE9A
cg27218767	2.56 × 10^−5^	3	142442934	TRPC1
cg13085232	2.58 × 10^−5^	1	10802080	CASZ1
cg26563242	2.72 × 10^−5^	1	46797699	
cg27170935	2.83 × 10^−5^	1	5221521	
cg03858387	2.87 × 10^−5^	15	25199164	SNRPN;SNURF
cg05435504	2.98 × 10^−5^	12	49251596	RND1
cg11908057	2.99 × 10^−5^	7	27171154	HOXA4
cg01316659	3.06 × 10^−5^	6	30418115	
cg27045794	3.13 × 10^−5^	1	187412747	
cg08727313	3.19 × 10^−5^	9	128734485	
cg06879681	3.21 × 10^−5^	8	1900524	ARHGEF10
cg10228283	3.23 × 10^−5^	1	153234387	LOR
cg03492327	3.28 × 10^−5^	14	57273276	OTX2
cg00733115	3.39 × 10^−5^	6	37105406	
cg04501323	3.58 × 10^−5^	1	235267609	
cg17619701	3.62 × 10^−5^	10	112610100	
cg21765224	3.64 × 10^−5^	20	34359771	PHF20
cg21528040	3.64 × 10^−5^	1	24195227	FUCA1
cg15895505	3.74 × 10^−5^	14	105903354	MTA1
cg14669919	3.79 × 10^−5^	11	65340482	FAM89B
cg04664999	3.85 × 10^−5^	19	14185985	
cg00499599	3.85 × 10^−5^	21	47706392	C21orf57;MCM3AP
cg17362661	3.87 × 10^−5^	2	100210490	AFF3
cg12010144	3.88 × 10^−5^	17	76733624	CYTH1
cg07568040	3.90 × 10^−5^	2	158454401	ACVR1C
cg18887769	3.95 × 10^−5^	14	22945181	
cg14251798	4.00 × 10^−5^	19	19545333	MIR640;GATAD2A
cg06836148	4.07 × 10^−5^	2	2957515	LINC01250
cg08920628	4.11 × 10^−5^	10	48354911	ZNF488
cg20827116	4.17 × 10^−5^	11	65627404	MUS81
cg11980004	4.20 × 10^−5^	7	1571105	MAFK
cg24383710	4.23 × 10^−5^	4	53916546	SCFD2
cg02645135	4.33 × 10^−5^	16	69516238	
cg13056505	4.34 × 10^−5^	1	156378014	C1orf61
cg22202891	4.35 × 10^−5^	2	216001968	ABCA12
cg09468051	4.38 × 10^−5^	4	41879262	
cg23053746	4.38 × 10^−5^	12	98811404	
cg13290331	4.40 × 10^−5^	13	49068807	RCBTB2
cg01660473	4.48 × 10^−5^	13	28395757	
cg01243529	4.49 × 10^−5^	3	194223220	
cg01183384	4.65 × 10^−5^	9	716332	KANK1
cg02281539	4.78 × 10^−5^	6	73273769	
cg20259534	4.88 × 10^−5^	15	40453036	BUB1B
cg18456621	4.93 × 10^−5^	17	80297270	
cg19359858	4.97 × 10^−5^	12	103667687	C12orf42

**Table 4:** Biological pathways overrepresented among genes of CpGs associated with PTG at *p* < 5 × 10^−5^ and *p* < 0.001.

**Pathways (*p* < 5 × 10^−5^ CpGs genes)**	**Number of genes**	***p*-value**	**FDR *p*-value**
ABC transporters	3	1.22 × 10^−4^	2.76 × 10^−2^
**Pathways (*p* < 0.001 CpGs genes)**	**Number of genes**	***p*-value**	**FDR *p*-value**
Phospholipase D signaling pathway	21	2.44 × 10^−5^	6.14 × 10^−3^
Axon guidance	23	4.42 × 10^−5^	6.14 × 10^−3^
EGFR tyrosine kinase inhibitor resistance	14	5.65 × 10^−5^	6.14 × 10^−3^
Morphine addiction	14	2.71 × 10^−4^	2.17 × 10^−2^
Dopaminergic synapse	17	5.05 × 10^−4^	2.17 × 10^−2^
Ras signaling pathway	25	5.16 × 10^−4^	2.17 × 10^−2^
AMPK signaling pathway	16	5.42 × 10^−4^	2.17 × 10^−2^
Inflammatory mediator regulation of TRP channels	14	6.57 × 10^−4^	2.17 × 10^−2^
Choline metabolism in cancer	14	6.57 × 10^−4^	2.17 × 10^−2^
GABAergic synapse	13	6.66 × 10^−4^	2.17 × 10^−2^
MAPK signaling pathway	29	8.63 × 10^−4^	2.51 × 10^−2^
Glutamatergic synapse	15	9.22 × 10^−4^	2.51 × 10^−2^
Autophagy	16	1.11 × 10^−3^	2.58 × 10^−2^
Thyroid hormone signaling pathway	15	1.11 × 10^−3^	2.58 × 10^−2^
Relaxin signaling pathway	16	1.31 × 10^−3^	2.83 × 10^−2^
Longevity regulating pathway	10	1.39 × 10^−3^	2.83 × 10^−2^
ErbB signaling pathway	12	1.59 × 10^−3^	3.06 × 10^−2^
Endocrine resistance	13	1.85 × 10^−3^	3.34 × 10^−2^
Proteoglycans in cancer	21	2.09 × 10^−3^	3.59 × 10^−2^
Endocytosis	24	2.35 × 10^−3^	3.83 × 10^−2^
Fc epsilon RI signaling pathway	10	2.83 × 10^−3^	4.13 × 10^−2^
Serotonergic synapse	14	2.85 × 10^−3^	4.13 × 10^−2^
Endocrine and other factor-regulated calcium reabsorption	8	2.91 × 10^−3^	4.13 × 10^−2^
Sphingolipid signaling pathway	14	3.62 × 10^−3^	4.91 × 10^−2^
Cell adhesion molecules (CAMs)	16	3.76 × 10^−3^	4.91 × 10^−2^

## Data Availability

The data is available upon request from the authors.

## Supplementary Materials

The following supporting information can be downloaded at: https://www.mdpi.com/article/10.3390/epigenomes9040039/s1, Table S1: The full EWAS results of the 845k CpGs assessed with changes in PTG between T_1_ and T_2_ are provided in Supplementary Table S1.
